# Impaired Skeletal Muscle Kynurenine Metabolism in Patients with Chronic Obstructive Pulmonary Disease

**DOI:** 10.3390/jcm8070915

**Published:** 2019-06-26

**Authors:** Harry R. Gosker, Gerard Clarke, Chiel C. de Theije, John F. Cryan, Annemie M. W. J. Schols

**Affiliations:** 1 NUTRIM School of Nutrition and Translational Research in Metabolism, Maastricht University Medical Centre+, Department of Respiratory Medicine, P.O. Box 616, 6200 MD Maastricht, The Netherlands; 2 APC Microbiome Ireland & Department of Psychiatry and Neurobehavioural Science, University College Cork, T12 YT20 Cork, Ireland; 3 APC Microbiome Ireland & Department of Anatomy and Neuroscience, University College Cork, T12 YT20 Cork, Ireland

**Keywords:** chronic obstructive pulmonary disease (COPD), skeletal muscle, mental health

## Abstract

Background: Loss of peripheral muscle oxidative phenotype, cognitive impairment, and depression are well-recognized systemic manifestations of chronic obstructive pulmonary disease (COPD). Kynurenine (KYN), known to be associated with disturbed mental health, can be metabolized in muscle by kynurenine aminotransferases (KAT) 1–4. These KATs are regulated by peroxisome proliferator-activated receptor gamma (PPARγ) coactivator-1α (PGC1α). We hypothesize that impaired PGC1α signaling in COPD is associated with reduced muscle KAT expression and increased KYN plasma levels. Methods: Retrospective collected and metabolically phenotyped muscle tissue and blood obtained from 29 well-characterized COPD patients and 15 healthy controls were analyzed. KYN was measured in plasma and KAT1–4 expression and major constituents of PGC1α signaling were assessed in quadriceps muscle biopsies. Results: Circulating KYN levels were increased in COPD. Furthermore, both gene and protein expression levels of KAT4 were reduced in muscle tissue from COPD patients. Finally, in the whole group (even when controlled for airflow obstruction) and in each subgroup separately, *KAT4* gene expression correlated significantly with constituents of the PGC1α signaling pathway. Conclusions: These data support our hypothesis that KYN plasma levels are elevated in COPD through impaired KYN clearance in muscle. Our findings show a pathway via which exercise training and/or nutritional modulation may improve physical and mental health in COPD patients.

## 1. Introduction

Extrapulmonary manifestations are prevalent in chronic obstructive pulmonary disease (COPD) and in particular skeletal muscle dysfunction and impaired mental health significantly contribute to a high disease burden [1,2]. Muscle wasting and dysfunction in COPD lead to reduced exercise tolerance, quality of life, and even survival [3]. Impaired cognition limits self-management skills, treatment compliance, and negatively affects health outcomes [4]. Depression in COPD patients has been associated with higher hospital admission rates, sleep disturbances, social isolation, and in severe cases, sometimes even suicidal behavior [5,6]. Lower limb muscle dysfunction is a consequence of muscle wasting and mitochondrial impairments [1,3]. The loss of muscle mitochondrial function and capacity has been attributed to the downregulation of key regulators of mitochondrial biogenesis; the peroxisome proliferator-activated receptors (PPARs) and master regulator PPARγ coactivator-1α (PGC1α) [3,7,8,9] which seems, at least partly, driven by inflammatory processes [10]. Mental health problems in COPD are reflected by a higher prevalence of cognitive impairment and depression [2,4,11]. The underlying mechanisms of cognitive impairment and depression in COPD remain unclear. Recently, elevated circulating kynurenine (KYN), a tryptophan metabolite, was found in COPD [12]. The authors proposed KYN as a novel biomarker of systemic inflammation, but this marker has also been associated with impaired cognition and depression in non-COPD subjects [13,14]. Moreover, KYN could be a link between muscle impairment and disturbed mental health in COPD: Circulating KYN can cross the blood–brain barrier and in the brain KYN metabolites may negatively affect mental health. Skeletal muscle can convert KYN into 3-hydroxykynurenine and kynurenic acid (KYNA; which in contrast to KYN cannot cross the blood–brain barrier) via the kynurenine aminotransferases (KAT) 1–4, thereby preventing KYN from entering the brain [15,16]. Interestingly, these KATs are regulated by PGC1α which, as mentioned above, has been found to be reduced in COPD. Furthermore, mice overexpressing PGC1α in muscle are resilient to developing depression [16]. Hence, we hypothesize that impaired muscle PGC1α signaling is associated with reduced KAT expression levels in skeletal muscle and with increased KYN plasma levels in patients with COPD.

## 2. Experimental Section

We tested our hypothesis using plasma and muscle tissue stored from a previously published study, in which reduced PGC1α signaling associated with loss of mitochondrial content was shown in vastus lateralis tissue obtained from 29 patients with clinically stable COPD versus 15 healthy controls (Table 1) [9]. The ethical review board of the Maastricht University Medical Centre+ approved the study (08-2-059) and written informed consent was obtained from all subjects. A detailed description of the methods can be found in the online supplementary material. Briefly, KYN and KYNA levels were determined in EDTA plasma by HPLC, as previously described [17]. Muscle tissue was homogenized, and KAT1–4 gene and protein expression levels were determined by RT-PCR (GeNorm-corrected, for primers see Table S1) and Western blotting (using primary antibodies anti-KAT1 (12156-1-AP; ProteinTech), anti-KAT2 (13031-1-AP; ProteinTech), anti-KAT3 (HPA026538; Atlas Antibodies), and anti-KAT4 (ARP43518-T100; AVIVA Systems Biology), respectively. Differences between COPD patients and controls were tested using Student’s *t*-test (with Levene’s test for equality of variances) or the Mann–Whitney U test as appropriate, and correlations were tested using Spearman’s correlation coefficient. A *p*-value < 0.05 was considered statistically significant.

## 3. Results

**Figure 1:**
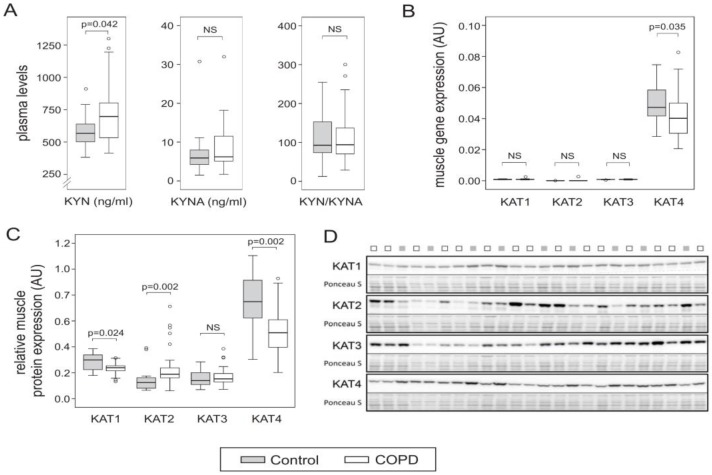
Boxplots representing (**A**) Kynurenine (KYN) and kynurenic acid (KYNA) plasma levels and ratio, (**B**) muscle kynurenine aminotransferases (*KAT*)*1–4* gene expression levels, and (**C**) muscle KAT1–4 protein expression levels. (**D**) Representative Western blot of KAT1–4 and corresponding PonceauS staining (300–20 kD range). NS, not significant.

KYN plasma levels were higher in COPD than in controls, but KYNA levels as well as the KYN–KYNA ratio were not different (Figure 1A). Although muscle *KAT1–3* gene expression was comparable between patients and controls, *KAT4* gene expression was significantly lower in COPD (Figure 1B). Also note that, in general, *KAT4* expression was much higher than *KAT1–3*. Relative muscle KAT1 and KAT3 protein levels were comparable between patients and controls, whereas KAT2 was higher in COPD (Figure 1C). However, in line with the gene expression data, KAT4 protein expression was significantly lower in COPD (Figure 1C).

**Figure 2:**
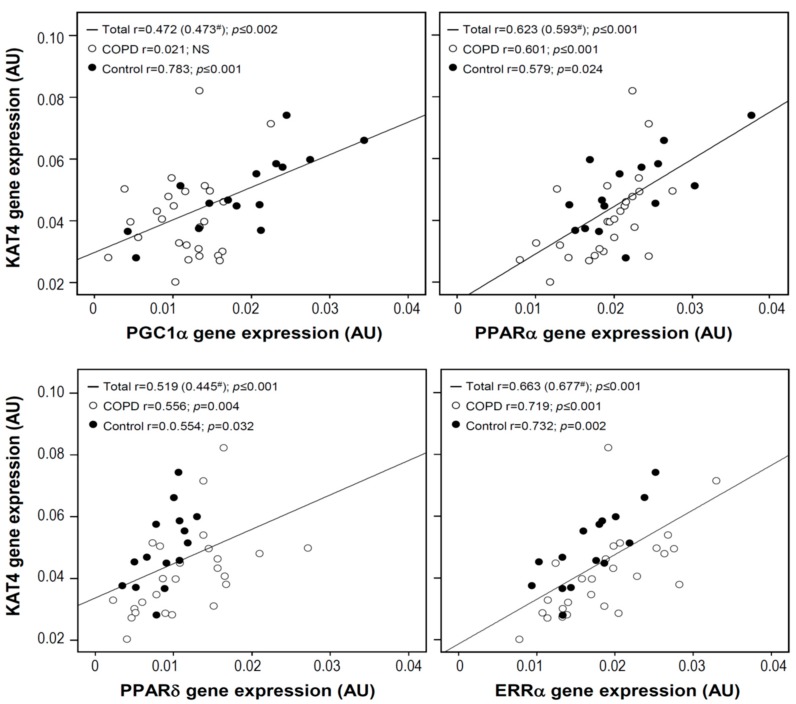
Spearman correlations between muscle *KAT4* gene expression and gene expression levels of peroxisome proliferator-activated receptor gamma coactivator-1-alpha (PGC1α), peroxisome proliferator-activated receptor alpha (PPARα), peroxisome proliferator-activated receptor delta (PPARδ), and estrogen-related receptor alpha (ERRα). ^#^ Partial correlation corrected for the forced expiratory volume in 1 s (FEV_1_) % predicted.

In muscle, *PGC1α* gene expression was positively correlated to *KAT4* in the total study population, as well as in the control group. In the study population as a whole, as well as in both COPD and control subgroups, there was also a strong positive correlation between muscle *KAT4* gene expression and that of the transcription factors PPARα, PPARδ, and estrogen-related receptor alpha (ERRα) (Figure 2).

## 4. Discussion

The observed elevated plasma KYN levels in COPD are in line with a recent report by Zinellu et al., who proposed KYN as a new biomarker of systemic inflammation in this disease [12]. However, based on the airflow obstruction measures, the patients studied by Zinellu et al. had considerably less severe COPD (FEV_1_ 80% predicted) as compared to the patients currently studied (FEV_1_ of 58% predicted). Possible mechanisms underlying elevated KYN levels could be enhanced production from tryptophan in the liver by corticosteroid-induced tryptophan-2,3-dioxygenase activity, or by systemic-inflammation induced indoleamine-2,3-dioxygenase activity [18]. However, the origin and implications of elevated KYN levels in COPD probably reach further than systemic inflammation alone, as the KYN pathway has also been implicated in the pathogenesis of depression and cognitive impairments and may play an important role in the crosstalk between muscle and the brain [15,16]. Using an animal model, Agudelo et al. showed that by regulating the KATs, muscle PGC1α signaling could be causally linked to attenuated KYN levels and subsequently to reduced depression [16]. Given that reduced muscle PGC1α–PPAR signaling in COPD is a consistent finding, we investigated muscle KAT expression and KYN blood samples in well-characterized samples from COPD patients versus healthy controls. 

We showed that KYN plasma levels are elevated in patients with COPD who are characterized by compromised muscle oxidative energy metabolism and decreased PGC1α signaling. Moreover, our data indicate that the decreased PGC1α signaling probably results in a perturbed KYN metabolism in skeletal muscle as well, which could contribute to the increased KYN plasma levels in these patients. We used RT-PCR to measure gene expression, which yields relative mRNA levels. However, based on the enormous difference between KAT4 and KAT1–3, KAT4 is most likely the most abundant of the four KATs in muscle tissue. In mice, KAT2 is even undetectable in muscle tissue [16]. Our data are completely in line with a recent report that studied muscle KAT expression in healthy males subjected to endurance exercise training [19]. The authors found that exercise training induced a particular upregulation of KAT4 along with an upregulation of PGC1α and PPARα. We also observed strong correlations between KAT4 and PPARα, PPARδ, ERRα, and their co-activator PGC1α. In addition, in mice with PGC1α being overexpressed or knocked down in muscle tissue specifically, KAT expression is increased and decreased, respectively, indicating the importance of PGC1α in KAT regulation [16]. In a recent study of cancer patients, an exercise-mediated improvement in depression was also linked to KYN metabolism [20]. We cannot rule out that other organs or tissues are involved in the elevated circulating KYN levels in COPD as well, but these findings suggest that exercise training, currently a core component of pulmonary rehabilitation to treat muscle dysfunction in COPD [21], by restoring muscle PGC1α signaling and KAT4 expression, might also be beneficial in the treatment of mental comorbidities. Indeed, two recent meta-analyses showed that pulmonary rehabilitation has beneficial effects on mental health in terms of cognition, anxiety, and depression in COPD patients [22,23]. Alternatively, or as an adjunct to exercise, nutritional supplements containing specific nutrients known to stimulate muscle PGC1α signaling (e.g., n-3 polyunsaturated fatty acids and polyphenolic compounds) may have pharmacologic potential to boost the KATs alongside mitochondrial capacity, especially in more severe COPD patients for whom the efficacy of aerobic exercise may be limited by their ventilatory impairment [24]. Future research should establish if KYN metabolism is the link between exercise-induced improvements in muscle and the brain in COPD and the actual potential of KYN metabolism in muscle as a direct target in the treatment of mental comorbidities in COPD.

There are some limitations to our study. Preexisting muscle and blood samples were used from a previous study [9] that did not aim to investigate mental health and therefore the observed alterations in KYN metabolism could not be linked to indices of cognition and depression in these COPD patients. Therefore, our findings must be confirmed in larger study populations in which mental health is extensively characterized. Furthermore, although we found a strong correlation between KAT4 expression and the PPAR–PGC1α pathway, future research should reveal whether the latter is indeed regulating KAT4 in muscle tissue—for example, by assessing KAT4 expression after modulating the PPAR–PGC1α pathway in experimental models. Also, we found neither reduced KYNA plasma levels in COPD nor an altered KYN–KYNA ratio. This would not have been unlikely based on the increased KYN levels and decreased KAT expression, since the KATs convert KYN into KYNA. It can be speculated that the anticipated drop in KYNA is masked by its attenuated clearance or elevated production elsewhere. There are alternative routes for KYNA production. For example, indole pyruvic acid can also be converted into KYNA in the presence of reactive oxygen species [25]. Interestingly, elevated reactive oxygen species levels, both systemically and locally in skeletal muscle, have consistently been found in COPD [3]. Nevertheless, the key point is that KYN can cross the blood–brain barrier whereas KYNA cannot and therefore the increased plasma KYN levels are most relevant in light of the crosstalk between muscle and the brain.

## 5. Conclusions

Our results indicate reduced KAT4 expression in the quadriceps muscle of COPD patients with elevated plasma KYN. Furthermore, our data suggest that impaired PGC1α signaling in the muscle might be involved. It can be speculated that skeletal muscle abnormalities in COPD, through impaired KYN metabolism, may negatively affect the mental health of these patients. Future studies using larger study populations are required to confirm our theory that altered KYN metabolism at the muscular level is indeed involved in impaired mental health in COPD. If so, then combined with our findings, this would potentially identify a novel pathway via which exercise training and/or nutritional modulation may not only improve physical performance but also mental health in COPD patients. 

## Figures and Tables

**Table 1:** Patient characteristics.

	Controls (*n* = 15)	COPD (*n* = 29)	
*Demographics*			
Age, years	65 (6)	65 (6)	NS
Sex, % men	60	55	NS
BMI, kg/m^2^	24.9 (3.3)	25.5 (3.6)	NS
*Pulmonary function*			
FEV_1_, % pred	113 (15)	58 (16)	*p* ≤ 0.001
FVC, % pred	120 (17)	104 (22)	*p* = 0.016
FEV_1_/FVC, %	74 (5)	45 (11)	*p* ≤ 0.001
DLCO, % pred	95 (19)	53 (18)	*p* ≤ 0.001
*Muscle Biopsy*			
*PGC1α* gene expression (AU)	0.019 (0.008)	0.011 (0.005)	*p* ≤ 0.001
ETC complex IV protein expression (AU)	5.11 (3.51)	2.71 (1.97)	*p* = 0.024
ETC complex V protein expression (AU)	0.83 (0.50)	0.57 (0.33)	*p* = 0.047

Abbreviations: COPD, chronic obstructive pulmonary disease; BMI, body mass index; DLCO, diffusion capacity of the lungs for carbon monoxide; FEV_1_, forced expiratory volume in 1 s; FVC, forced vital capacity; ETC, electron transport chain (mitochondrial proteins); NS, not significant; % pred, % predicted. Data are presented as mean (SD). Adapted from [9].

## Supplementary Materials

The following are available online at https://www.mdpi.com/2077-0383/8/7/915/s1, detailed additional methods description, Table S1: Primer sequences.
