# Supplementary material for: Impaired Skeletal Muscle Kynurenine Metabolism in Patients with Chronic Obstructive Pulmonary Disease
Source: J Clin Med. 2019 Jun 26;8(7):915. doi: 10.3390/jcm8070915 (PMC6678819; doi:10.3390/jcm8070915)
Supplement: Supplementary file 1 [file jcm-08-00915-s001.pdf]

Journal of
Clinical Medicine

MDPI

Supplemental Material

Impaired Skeletal Muscle Kynurenine Metabolism in Patients with Chronic Obstructive Pulmonary Disease

Running head: Impaired muscle kynurenine metabolism in COPD

Harry R. Gosker (Dr.)[1]*, Gerard Clarke (Dr.)[2], Chiel C. de Theije (Dr.) [1], John F. Cryan (Prof. Dr.)[3], Annemie M.W.J. Schols (Prof. Dr.)[1]

[1] NUTRIM School of Nutrition and Translational Research in Metabolism, Maastricht University Medical Centre+, Department of Respiratory Medicine, Maastricht, The Netherlands

[2] APC Microbiome Ireland & Department of Psychiatry and Neurobehavioural Science, University College Cork, Cork, Ireland

[3] APC Microbiome Ireland & Department of Anatomy and Neuroscience, University College Cork, Cork, Ireland

Methods

For this study we used pre-existing plasma and muscle tissue obtained during a previously published study in which reduced PGC1α-signaling associated with loss of mitochondrial content was shown in *vastus lateralis* tissue obtained from 29 patients with clinically stable mild-to-moderate COPD *versus* 15 healthy controls.[1] Patients were recruited from the outpatient clinic of the Maastricht University Medical Center (MUMC+, Maastricht, the Netherlands) and via advertisements in local newspapers. Patients were excluded if they were on long-term oxygen therapy, used oral prednisolone, or had an acute exacerbation of symptoms with hospital admission in the previous 8 wk and rehabilitation in the previous 6 months. Patients with a known comorbidity (e.g. diabetes, recent cardiovascular event, inflammatory bowel disease, obstructive sleep apnea, thyroid disease, and cancer) that could potentially interfere with study outcome parameters were carefully excluded. Healthy controls were recruited via advertising in local newspapers. The absence of these diseases in the healthy subjects was verified through history-taking by a physician and pulmonary function tests to verify the absence of airflow limitation. Written, informed consent was obtained from all subjects and the ethical review board of the MUMC approved the study (08-2-059). The trial was registered at http://www.trialregister.nl as NTR1402.

Muscle Biopsy

Biopsies of the quadriceps muscle (*vastus lateralis*) from the dominant leg were obtained using a needle biopsy technique.[2] Tissue was snap-frozen in liquid nitrogen and stored at -80°C for gene and protein expression analyses.

RNA Extraction and RT-qPCR Analysis

Muscle tissue (10–30 mg) was homogenized in Denaturation Solution (ToTALLY RNA™ Kit; Ambion Ltd., Foster City, CA, USA) using a Polytron PT 1600 E (Kinematica AG, Littau/Luzern, Germany) and RNA extracted according to the supplier's protocol, followed by genomic DNA removal and clean-up with the RNeasy Mini Kit with RNase-free DNase (Qiagen, Venlo, the Netherlands). After elution, RNA concentration was determined using a spectrophotometer (NanoDrop ND-1000, Isogen Lifescience, IJsselstein, the Netherlands) and integrity verified for a selection of samples by gel electrophoresis and on a Bioanalyzer (Agilent Technologies, Amstelveen,

the Netherlands). 400ng RNA was reverse transcribed to cDNA with anchored oligo(dT) primers according to the supplier's protocol (Transcriptor First Strand cDNA Synthesis kit, Roche Diagnostics, Woerden, the Netherlands). RT-qPCR primers (table S1) were designed based on Ensembl transcript sequences and ordered from Sigma Genosys (Zwijndrecht, the Netherlands). The distance of the PCR primers to the 3' termini was within the limit of the RT kit. The distance to the 3' termini of the KAT1-4 primers was comparable. qPCR reactions contained Sensimix SYBR & ROX (Bioline, Waddinxveen, the Netherlands) and primer mix and were run in a 384 well white opaque plate on a LightCycler 480 system (Roche, Almere, the Netherlands). Melting curves were analysed to verify specificity of the amplification, and relative quantity of the targets was assessed by LinRegPCR software (v2014.8.1), which corrects for any potential differences in PCR efficiency for each target. GeNorm (Primerdesign, Southampton, USA) correction was used to normalize expression of the target genes (housekeeping genes listed in e-Table 1).[3] Specific sample measurements were excluded when individual PCR efficiency was deviating from average PCR efficiency.

Table 1. Primer sequences.

	Gene Name	Forward Primer	Reverse Primer	ENSEMBL ID
	Targets			
PGC1a	PPARgamma coactivator 1 beta	GACCAGTGCTACCTGAGAGAGACTT	GCTCGGCTCGGATTTCCT	ENSG00000109819
PPARa	Peroxisome Proliferator Activated Receptor alpha	CAGAACAAGGAGGCGGAGGTC	AGGTCCAAGTTTGCGAAGC	ENSG00000186951
PPARg	Peroxisome Proliferator Activated Receptor gamma	CGGGCCCTGGCAAAAC	AAGATCGCCCTCGCCTTT	ENSG00000132170
ERRa	Estrogen related receptor alpha	GCGGCTGGAGCGAGAGGAG	CTCGGCATCTTCGATGTGCAC	ENSG00000173153
KAT1	Kynurenine aminotransferase 1	CCTGACTTGCCTGGAGCTGT	GACAGGGATGGCCACCAA	ENSG00000171097
KAT2	Aminoadipate aminotransferase	TTCAGCTTCTCCAGAACAGATGG	GCCACCATGCCCAACCTAGT	ENSG00000109576
KAT3	Kynurenine aminotransferase 3	CCATCCCCGTTTCAGCATT	GCAGCATCCAGTGTGCTGTCT	ENSG00000137944
KAT4	Glutamic-oxaloacetic transaminase 2	GCAACACATCACCGACCAAA	ATGCGGCCATCTTTTGTCAT	ENSG00000125166
	Housekeepers			
B2m	beta-2-microglobulin	CTGTGCTCGCGCTACTCTCTCTT	TGAGTAAACCTGAATCTTTGGAGTACG C	ENSG00000166710
Ppia	Cyclophilin	CATCTGCACTGCCAAGACTGA	TTCATGCCTTCTTTCACTTTGC	ENSG00000196262
Rpl13A	Ribosomal protein 13A	CCTGGAGGAGAAGAGGAAAGAGA	TTGAGGACCTCTGTGTATTTGTCAA	ENSG00000142541
Rplp0	Large ribosomal protein	TCTACAACCCTGAAGTGCTTGATATC	GCAGACAGACACTGGCAACATT	ENSG00000089157
ALAS1	5-Aminolevulinate synthase	CTGCAAAGATCTGACCCCTC	CCTCATCCACGAAGGTGATT	ENSG00000023330
ACTB	Beta Actin	AAGCCACCCCACTTCTCTCTAA	AATGCTATCACCTCCCCTGTGT	ENSG00000075624
GAPDH	Glyceraldehyde-3-phosphate dehydrogenase	GCACCACCAACTGCTTAGCA	TGGCAGTGATGGCATGGA	ENSG00000111640
GUSB	Beta-glucuronidase	CTCATTTGGAATTTTGCCGATT	CCGAGTGAAGATCCCCTTTTTA	ENSG00000169919
HMBS	Hydroxymethyl-bilane synthase	GGCAATGCGGCTGCAA	GGGTACCCACGCGAATCAC	ENSG00000256269
HPRT1	Hypoxanthine phosphoribosyl-transferase 1	TGACACTGGCAAAACAATGCA	GGTCCTTTTCACCAGCAAGCT	ENSG00000165704

UBC	Ubiquitin C	ATTTGGGTCGCAGTTCTTG	TGCCTTGACATTCTCGATGGT	ENSG00000150991
YWHAZ	Tyrosine 3-monooxygenase/ tryptophan 5-monooxygenase activation protein, zeta polypeptide	ACTTTTGGTACATTGTGGCTTCAA	CCGCCAGGACAAACCAGTAT	ENSG00000164924

Western Blotting

Muscle tissue (10–20 mg) was crushed in liquid nitrogen and homogenized in 400µl IP lysis buffer (50mM Tris, 150mM NaCl, 10% glycerol, 0.5% Nonidet P40, 1mM EDTA, 1mM Na_3VO_4, 5mM NaF, 10mM β-glycerophosphate, 1mM $Na_4O_7P_2$, 1mM dithiothreitol, 10µg/ml leupeptin, 1% aprotenin, 1mM PMSF, pH 7.4) with a Polytron PT 1600 E (Kinematica). After homogenization, samples were incubated for 15 minutes on a rotating wheel at 4°C and spun for 30 minutes at maximum speed (20817×*g*) in a centrifuge cooled to 4°C. Supernatant was aliquoted, snap-frozen and stored without sample buffer at −80°C until analysis.

Protein concentration in lysates was determined using the bicinchoninic acid assay (Pierce, Thermo Fisher Scientific, Breda, the Netherlands). For western blot analysis, aliquots were supplemented with 4× sample buffer (250mM Tris-HCl pH6.8, 8% (w/v) sodium dodecyl sulfate, 40% (v/v) glycerol, 0.4M dithiothreitol, 0.02% (w/v) bromophenol blue) and kept on ice. Per sample, 10 µg boiled protein was separated on gel (4-12% Bis-Tris XT gel, Criterion, Bio-Rad) with XT MOPS running buffer (Bio-Rad). Proteins were transferred to a 0.45µm nitrocellulose membrane (Biorad, Veenendaal, the Netherlands) in transfer buffer (25mM Tris, 192mM glycine, 20% (v/v) methanol) by electrophoresis. After transfer, membranes were blocked from nonspecific protein binding with blocking solution, which contains 3% (w/v) non-fat dry milk (Campina, Eindhoven, the Netherlands) in Tris-buffered saline with Tween20 (TBST; 25mM Tris, 137mM NaCl, 2.7mM KCl, 0.05% (v/v) Tween20, pH 7.4), for one hour at room temperature, followed by incubation in primary antibody solution overnight at 4°C (rabbit anti-KAT1 (12156-1-AP; ProteinTech), anti-KAT2 (13031-1-AP; ProteinTech), anti-KAT3 (HPA026538; Atlas Antibodies), and anti-KAT4 (ARP43518_T100; AVIVA Systems Biology) diluted 1:1000 in 1% BSA in TBST. Membranes were incubated in secondary antibody solution (peroxidase-labeled horse anti-rabbit IgG (PI-1000, Vector Laboratories, Burlingame, CA, USA) diluted 1:10000 in blocking solution) for one hour at room temperature before incubation with enhanced chemiluminescence substrate (Pierce SuperSignal West PICO Chemiluminescent Substrate; Thermo Fisher Scientific). Protein bands were detected using a LAS-3000 Luminescent Image Analyzer (Fujifilm, Tokyo, Japan). PonceauS staining was used to control and correct for protein loading (in figure 1D the 300-20 kD range was cropped and downscaled vertically).

Blood Analyses

Kynurenine (KYN) and kynurenic acid (KYNA) were determined as previously described by Clarke *et al.*[4] Briefly, EDTA plasma samples were spiked with internal standard (3-Nitro-l-tyrosine) prior to being deproteinised by the addition of 20 µl of 4 M perchloric acid to 200 µl of sample. Samples were centrifuged at 21,000g on a Hettich Mikro 22R centrifuge (AGB, Dublin, Ireland) for 20 min at 4 °C and 100 µl of supernatant transferred to a HPLC vial for analysis on the HPLC system (UV and FLD detection). All samples were injected onto a reversed phase Luna 3 µm C18 (2) 150 × 2 mm column (Phenomenex), which was protected by KrudKatcher disposable pre-column filters (Phenomenex) and SecurityGuard cartridges (Phenomenex). The mobile phase consisted

of 50 mM acetic acid and 100 mM zinc acetate with 3% (v/v) acetonitrile and was filtered through Millipore 0.45-µm HV Durapore membrane filters (AGB) and vacuum degassed prior to use. Compounds were eluted isocratically over a 30-min runtime at a flow rate of 0.3 ml/min after a 20-µl injection. The column was maintained at a temperature of 30 °C, and samples/standards were kept at 8 °C in the cooled autoinjector prior to injection. The fluorescent detector was set at an excitation wavelength of 254 nm and an emission wavelength of 404 nm. The UV detector was set to 330 nm and kynurenines were identified by their characteristic retention times as determined by standard injections which were run at regular intervals during the sample analysis. Analyte:Internal standard peak height ratios were measured and compared with standard injections, and results were expressed as nanogram per milliliter of plasma.

References

1. van den Borst, B.; Slot, I.G.; Hellwig, V.A.; Vosse, B.A.; Kelders, M.C.; Barreiro, E.; Schols, A.M.; Gosker, H.R. Loss of quadriceps muscle oxidative phenotype and decreased endurance in patients with mild-to-moderate COPD. *Journal of applied physiology* **2013**, *114*, 1319-1328, doi:10.1152/japplphysiol.00508.2012.
2. Bergstrom, L. Muscle electrolytes in man. Determination by neutron activation analysis on needle biopsy specimens. A study on normal subjects, kidney patients, and patients with chronic diarrhea. *Scand J Clin Lab Invest* **1962**, *68*, 1-110.
3. Vandesompele, J.; De Preter, K.; Pattyn, F.; Poppe, B.; Van Roy, N.; De Paepe, A.; Speleman, F. Accurate normalization of real-time quantitative RT-PCR data by geometric averaging of multiple internal control genes. *Genome Biol* **2002**, *3*, RESEARCH0034.
4. Clarke, G.; Fitzgerald, P.; Cryan, J.F.; Cassidy, E.M.; Quigley, E.M.; Dinan, T.G. Tryptophan degradation in irritable bowel syndrome: evidence of indoleamine 2,3-dioxygenase activation in a male cohort. *BMC Gastroenterol* **2009**, *9*, 6, doi:10.1186/1471-230X-9-6.
